# Sintomatología inicial de la infección por SARS-CoV-2 en el personal sanitario: ¿qué hemos aprendido?

**DOI:** 10.1016/j.aprim.2020.08.009

**Published:** 2020-12-19

**Authors:** Marta Bonet Beltrán, Lourdes Villarte Farré, Esther Barbé Illa, Pere Godoy

**Affiliations:** a Servicio de Oncología Radioterápica, HU Arnau de Vilanova, Lleida, España; b Diplomada en Salud Pública por el Institut d’Estudis de la Salut, Barcelona, España; c Servicio de Prevención de Riesgos Laborales, Institut Català de la Salut Lleida, Lleida, España; d Cap del Servei de Vigilància Epidemiològica i Resposta a Emergències de Salut Pública a Lleida i Alt Pirineu i Aran, Lleida, España IRBLleida (Institut de Recerca Biomèdica de Lleida. Fundació Doctor Pifarré)

*Sr. Editor:*

Un 24,1% de los casos declarados a la Red Nacional de Vigilancia Epidemiológica (RENAVE) desde el inicio de la alerta por SARS-CoV-2 hasta el 10 de marzo fueron profesionales de la salud^1^. Son muchas las medidas que se están adoptando en la actualidad con la finalidad de reducir el impacto en el sistema sanitario de las nuevas oleadas de infecciones, siendo clave el poder determinar un patrón sintomático inicial de la infección por SARS-CoV-2 con la finalidad de permitir la detección precoz y frenar la cadena de transmisión de la enfermedad en el medio sanitario.

Hemos realizado un estudio descriptivo a partir de los datos recogidos en el momento del contacto telefónico del trabajador con la Unidad de Prevención de Riesgos Laborales del Hospital Universitario Arnau de Vilanova de Lleida durante las primeras 4 semanas de atención y seguimiento de los casos comunicados —a partir del 17 de marzo—, computando un total de 203 profesionales sanitarios procedentes de 3 centros hospitalarios, 2 centros residenciales y 28 áreas básicas de salud de la región sanitaria de Lleida. Hemos analizado la sintomatología inicial referida por los trabajadores que dieron resultado positivo para SARS-CoV-2 mediante técnica PCR de muestras obtenidas por frotis nasofaríngeo. Los trabajadores con PCR negativa, así como los completamente asintomáticos testados a partir de un estudio de contactos, han sido excluidos del análisis.

De los 203 trabajadores con resultado positivo, hemos podido valorar la sintomatología inicial de 101. La mediana de edad fue de 43 años (media: 43,2; DE = 1), con un 74,3% de mujeres y un 25,7% de varones. Los profesionales mayormente afectados fueron los diplomados sanitarios de enfermería (n = 33; 32,67%), seguido del personal facultativo (n = 26; 25,7%), de técnicos en curas auxiliares de enfermería (n = 17; 16,8%), personal administrativo (n = 14, 13,9%), celadores (n = 8; 7,9%) y otros técnicos sanitarios superiores (n = 3; 3,0%). La sintomatología más frecuente fue de tipo inespecífico (mal estado general, artromialgias y/o astenia), seguida de tos, febrícula, cefalea, odinofagia y fiebre de más de 38 °C (tabla 1). Los trabajadores que describieron anosmia y ageusia fueron únicamente mujeres. Además, solo 7 presentaron síntomas típicos de infección respiratoria aguda (tos y fiebre alta), siendo la combinación de síntomas más frecuente el mal estado general y la tos (28%).

**Tabla 1:** Sintomatología descrita al diagnóstico por los profesionales sanitarios con infección COVID-19

Síntoma	n	%
Mal estado general/artromialgias/astenia	60	59,4
Tos	58	57,4
Febrícula	44	43,6
Cefalea	38	37,6
Odinofagia	19	18,8
Fiebre > 38 °C	18	17,8
Dolor torácico/disnea	9	8,9
Diarrea	9	8,9
Anosmia	7	6,9
Ageusia	7	6,9
Congestión nasal	7	6,9
Dolor abdominal	4	4,0
Náuseas	2	2,0
*Rash* cutáneo	1	1,0

En un contexto en el que en nuestro centro ya se estaban aplicando medidas de contención, este estudio ha permitido constatar la presencia de sintomatología inicial inespecífica en casi el 60% de los casos. Subrayamos asimismo que, respecto a otros patrones sintomáticos en la población general, los trabajadores sanitarios en nuestro estudio parecen presentar mayor clínica de cefalea y/o diarrea, y solo constatamos fiebre alta en el 17,5% de los mismos y tos en un 57,4%. Por otra parte, la cefalea, que ha sido referida por un 37,6% de nuestros trabajadores sanitarios con test PCR^+^ (que solo se describe en un 13,6% de los pacientes de la misión de la OMS en China), es un síntoma que algunos autores atribuyen al propio uso de los equipos de protección^2^.

Este espectro de sintomatología inicial inespecífica se asemeja al recientemente descrito en una serie de 48 trabajadores sanitarios en EE. UU.^3^ en el que los síntomas más comunes fueron la tos (50,0%), la fiebre (41,7%) y las mialgias (35,4%), también con una mediana de edad de 43 años y afectando a un 77,1% de mujeres. Entre los trabajadores que no presentaron sintomatología típica los síntomas más comunes fueron los escalofríos, las mialgias, la coriza y el malestar general. Los autores calculan que, si se hubieran incluido los escalofríos y las mialgias en el cribado, la detección de casos habría aumentado al 89,6%. Nuestro porcentaje de trabajadoras con PCR^+^ mujeres concuerda con los datos notificados a la RENAVE, si bien podría estar condicionado por el perfil de género de los profesionales sanitarios. Según los datos del INE en el año 2018, el 84,2% de los diplomados universitarios de enfermería, el 71,6% de los farmacéuticos y el 51,1% de los médicos, fueron mujeres^4^.

En un momento en el que la capacidad de respuesta del sistema sanitario depende entre otros factores de la salud de sus trabajadores, es de gran importancia reconocer de forma precoz cuál es el patrón sintomático al diagnóstico que se asocia con un resultado PCR^+^. Este estudio contribuye en la descripción del patrón sintomático más frecuente en los profesionales sanitarios. Síntomas como mal estado general, artromialgias, astenia, tos, febrícula y/o cefalea deberían ser considerados para el despistaje precoz de la infección.

## Financiación

El presente trabajo no presenta ninguna relación económica ni personal que haya podido sesgarlo.
